# Practical specification and application of implementation strategies to cognitive-behavioral research and practice

**DOI:** 10.3389/fmed.2026.1625471

**Published:** 2026-08-07

**Authors:** Robert E. Brady, Renee Pepin, Martha L. Bruce

**Affiliations:** Geisel School of Medicine at Dartmouth, Dartmouth-Hitchcock Medical Center, Lebanon, NH, United States

**Keywords:** cognitive-behavioral therapy, dissemination, evidence-based practice, implementation, strategies

## Abstract

Cognitive-behavioral therapy (CBT) is a first-line intervention for anxiety and mood disorders, yet its reach has not been fully realized due to incomplete implementation in routine community practice. The field of implementation science has the potential to improve integration of CBT in the settings and populations that most stand to benefit. Numerous strategies have been proposed to improve implementation, but are not consistently specified in the research, particularly in CBT research and practice. The present narrative review is intended to demonstrate to mental health clinicians, researchers, and policy-makers the ways in which implementation strategies can be utilized and specified to improve implementation of CBT, using a previously developed implementation strategy classification scheme as a guide. We describe the categories of strategies, provide exemplars of their use in CBT research and implementation efforts, and summarize the implications of each category type for CBT practice and research. We conclude with a hypothetical implementation study illustrating optimal use and specification of these strategies targeting implementation of CBT for OCD.

## Introduction

Cognitive-behavioral therapy (CBT) is a first-line intervention for anxiety and depressive disorders (1). A review of meta-analyses shows response rates of 38–77% for patients receiving CBT for anxiety disorders and 51–87% for depression (2). Yet, the demonstrated success of CBT within clinical trials has outstripped the capability of health systems and community-based providers to successfully implement the intervention in clinical settings (3). There is some evidence to suggest that opinions toward evidence-based practices (EBPs) are positive (4), but less evidence to suggest that the actual practice trend has changed (5, 6). In the absence of dedicated work to further implementation, CBT may remain a highly efficacious intervention that is inaccessible to the populations that it is intended to help.

In an update to the criteria for designating an intervention as an EBP, Tolin and colleagues proposed the addition of a new criterion in which an intervention must demonstrate *effectiveness* in at least one trial to receive a “very strong” recommendation (7). This marked a shift in thinking about EBPs wherein the ability of an intervention to demonstrate effectiveness in a real-world setting beyond the tightly controlled context of the laboratory is necessary to designate a practice “evidence-based.” The emphasis on effectiveness in routine care to determine the evidence-based nature of an intervention aligns with the goal of implementation science to increase the likelihood that EBPs are implemented in routine clinical practice.

Implementation researchers have heeded the call to study how best to disseminate and implement CBT for anxiety and other psychiatric conditions. This has led to a large body of research demonstrating effective implementation of EBPs, especially CBT for anxiety and mood disorders, though largely in highly structured medical systems and less so in routine community practice (8–10). However, studies investigating dissemination and implementation of CBT for anxiety disorders use inconsistent terminology to characterize implementation approaches. This limits synthesis across studies and may impede successful implementation. Inconsistent language around implementation strategies limits the ability of researchers and clinicians to effectively synthesize findings across studies that would otherwise demonstrate the evidence for implementation of a given intervention, sometimes referred to as the “Tower of Babel” in implementation science (11).

Proctor, Powell, and McMillen recognized the need to improve communication of implementation strategies across the field of implementation science by “specifying and categorizing” approaches (12). They advocated for (1) naming, (2) defining, and (3) operationalizing the strategies across multiple domains of specification (e.g., actors, targets, dose, etc.). Leeman and colleagues subsequently proposed a classification system for implementation strategies based on Proctor et al.’s suggestion to categorize on the basis of actors and action targets (13). This classification scheme combines the actor types from the Interactive Systems Framework (ISF) with action targets specified in the Consolidated Framework for Implementation Research (CFIR) (14–16). The ISF includes the Synthesis and Translation, Delivery, and Support systems.

The ISF defines the Synthesis and Translation System as actions leading to the compilation of the research information and translation into a format usable to those actors involved in the delivery of an intervention. Accordingly, the Delivery System includes the actions leading to the actual implementation of the intervention. The Support System include actions that facilitate implementation through the administration of resources aimed at supporting individuals and organizations intervening in a population.

The CFIR domains include aspects of the intervention (e.g., CBT), inner setting (e.g., clinic culture or structure), outer setting (e.g., clinical practice guidelines, geographic practice norms), individuals (e.g., provider beliefs about CBT, readiness to implement), and process (e.g., engaging clinical leadership, plan for training clinicians). The resulting classes or categories of strategies include: (1) dissemination, (2) implementation process, (3) integration, (4) capacity-building, and (5) scale-up (13).

This narrative review and commentary highlights examples and opportunities to tie implementation strategies for improving delivery of CBT for anxiety and mood disorders to an established categorical system. While several reviews cover implementation science applied to CBT broadly or within discrete disorders (17–19), the present narrative review is narrower, focusing on application of a classification system as a guiding structure for grouping instances of those strategies in the CBT literature. We chose the Leeman et al. classification system because it achieves the goal of succinctly grouping strategies in a way that can be efficiently communicated, aligned with the recommendations to specify and categorize strategies (14). We briefly describe each of the five forms of implementation approaches articulated by Leeman et al. before identifying an example of each approach from the available CBT literature. Each study is exemplary of the application of the category of implementation strategy. We then describe the use of the category in CBT research and practice and describe opportunities to expand on this work in subsequent research. Finally, we summarize efforts to improve implementation of CBT consistent with the Leeman et al. framework, highlight opportunities for increased specification of strategies within these categories, and advocate for an agenda to promote improved implementation of CBT using a fictional implementation effort to depict the application and identification of categories of implementation strategies.

## Public health significance

Cognitive-behavioral therapy is an evidence-based practice for several common mental disorders, but suffers from limited reach within populations in need of intervention. The limited application of cognitive-behavioral therapy for these population likely results from incomplete utilization of dissemination and implementation strategies. This narrative review presents a concise categorization of these strategies that could be readily employed by researchers, clinicians, and policy-makers to more effectively extend the reach of cognitivebehavioral therapy.

## Exemplar study identification

We conducted a broad survey of the CBT literature, including “third-wave” behavior therapies, to identify exemplar studies for the strategy categories. Because the goal of this manuscript was conceptual illustration rather than exhaustive evidence synthesis, we used purposive literature sampling to identify studies exemplifying the implementation strategy categories proposed by Leeman et al. We identified relevant articles through targeted database searches (including PubMed and PsycInfo), review of reference lists, and iterative discussion among the authors regarding conceptual fit within the proposed framework. The sampling search was initially designed and conducted in December 2020 for a related project; the search was revised and repeated in April 2025 for the current manuscript.

Studies were classified within a strategy category if the study procedures or implementation strategy was enacted by an ISF actor type (synthesis/translation, delivery, or support system) and specified the action target level (intervention, inner setting, outer setting, individuals, or process). We did not narrow categorization to the individual determinants in each action target since the range of determinants varied too greatly to categorize effectively. When an exemplar study included multiple strategies that could belong to multiple categories, we used it as category exemplar aligned with its primary focus in the manuscript based on author consensus.

## Categories and use of implementation strategies in CBT research and practice

### Dissemination strategies

The National Institutes of Health define dissemination as the “targeted distribution of information and intervention materials to a specific public health, clinical practice, or policy audience” with “intent is to understand how best to communicate and integrate knowledge and the associated evidence-based interventions” and differentiates this from implementation which is defined as “the use of strategies to adopt and integrate evidence-based health interventions into clinical and community settings to improve individual outcomes and benefit population health.” (20) That is, dissemination strategies are pre-implementation procedures intended to increase the likelihood of subsequent successful implementation.

Leeman et al. define dissemination strategies as “any action or set of actions that target public health and healthcare decision-makers’, clinicians’, and other staffs’ awareness, knowledge, and intention to adopt an evidence-based intervention.” In other words, dissemination strategies are efforts intended to increase the interest and engagement of non-patient stakeholders with a given intervention with the downstream goal of increasing the availability of the practice within the designated setting. Dissemination efforts in healthcare generally include providing information about best practices to providers through means that are most effective at reaching the target audience. From an implementation science perspective, this requires research-based knowledge of (1) what information is most effective at changing provider behaviors and (2) how to deliver that information most effectively. This is akin to marketing in business and other non-healthcare settings where the goal is to target a message to the right audience through the right channels at the right time.

#### Dissemination strategies in CBT

Consistent with the ISF model, dissemination strategies can be enacted by each level of actor. The Cochrane Collaboration is a clear example of dissemination strategies being used within the synthesis and translation level. Similarly, professional organizations function within the synthesis and translation system through the development of clinical practice guidelines. Notably, they can also comprise the support system by providing continuing education and ongoing consultation, such as that provided by Division 12 of the American Psychological Association or the Association for Behavioral and Cognitive Therapies. Finally, dissemination strategies are utilized at the level of the delivery system when an organization hosts presentations and in-service trainings on evidence-based interventions. However, dissemination of training and practice materials that are a poor fit for the targeted settings hamper efforts to improve the implementation of CBT. If successful dissemination is, in part, a product of effective marketing, then adapting information to preferences of the providers, setting, and institutional culture is likely to improve implementation via improved dissemination.

A dissemination effort conducted by Landes, Smith, and Weingardt provides an example of the process of targeted dissemination strategies (21). In this project, the authors developed a virtual community of practice (VCoP) with the goal of increasing implementation of dialectical behavior therapy (DBT). A VCoP is a method of facilitating communication and collaboration among a group of providers of a given intervention through Internet or other technologically-assisted means. The VCoP consisted of a website developed to host resources relevant and useful to the practice of DBT and discussion forums that would help implement and sustain DBT practice.

Importantly, the study team engaged in a planned dissemination effort to increase uptake of the VCoP using several strategies. After making the website available to any provider within the Veterans Affairs (VA) clinical system, the team contacted providers who had previously provided information to help develop the website and encouraged these individuals to forward the website information to their colleagues. They then advertised the website on a series of listservs relevant to DBT practice (e.g., VA EBP coordinator listserv, international DBT listserv), created an e-mail signature banner to create visibility in communications, advertised on the VA Mental Health Services website and in a VA-distributed e-mail newsletter, promoted the website during professional conference presentations, and worked with DBT training companies to refer trainees to the website. These methods of dissemination provide a demonstration of using multiple strategies to facilitate “targeted distribution” of the intervention information delivered through a website.

#### Critical issues for dissemination strategies in CBT

Cognitive-behavioral researchers and practitioners could improve their use of dissemination strategies in several ways, such as refining the language used in promotion of EBP for mental health providers, consistent with the notion of “pull” from the push-pull concept of adoption (22). Although science-minded researchers are committed to the promotion of EBPs some community providers may be less concerned about the evidence base for a practice than about practices that are “effective” and have the potential to engage patients (23). Additionally, some studies demonstrate that a substantial proportion of providers view the results of randomized controlled trials or other empirical evaluation of interventions negatively and associate it with academicians disconnected from the realities of health service provision (24, 25). Reducing the use of the phrase “evidence-based practice” or “empirically-supported therapies” in promotional materials does not of course reduce the evidence-based and empirically-supported nature of the promoted interventions. CBT dissemination efforts may be more effective when the emphasis is placed on the positive benefits of an intervention to patients more than scientific rigor. Doing so does not diminish the rigor, but rather increases the salience of the language to clinicians who may value patient benefit over the rigor of methods.

Researchers evaluating the effectiveness of CBT interventions may also need to attend to the use of highly technical jargon when developing and subsequently publishing the resulting manuals and other educational materials derived from those studies. Intervention materials are often written in a manner that speaks to those at the researcher’s same academic knowledge level rather than adjusting for the knowledge level possessed by those that are actually delivering the intervention (26). For example, an EBP manual that presents the rationale for delivering exposure therapy using a detailed description of causal theories of fear extinction may be factually correct, but miss the intended audience by using language that differs substantially from their prior training. Accordingly, if an intervention is to be delivered by clinicians or lay providers lacking the level of specialization possessed by the intervention developer, then tailoring the dissemination materials to fit the technical knowledge level with approachable language and terminology of the intended clinician may improve implementation.

Finally, the support system attempting to improve knowledge and utilization of CBT might consider alternative routes for dissemination that take advantage of provider preferences and capability. The study conducted by Landes et al. reflects the recognition that effective dissemination requires alignment with provider preferences. In this case, that meant using a mix of existing online channels that were already subscribed to by the target audience and developing a community of practice to enhance dissemination. This method of engaging clinicians with information about an EBP is likely to subsequently improve implementation. Similarly, using direct-to-consumer marketing of psychosocial interventions has the potential to increase the demand for CBT and other EBPs by encouraging providers to deliver what patients demand (27, 28). That assumes that the evidence for those interventions has generally reached a level of confidence that such marketing is ethically justified.

### Implementation process strategies

While dissemination strategies may appear obvious as components of a successful implementation effort, implementation process strategies may be less familiar to CBT researchers and clinicians. Implementation process refers to those procedures (e.g., strategies) aimed to make implementation more readily achievable within a setting and are agnostic to a particular intervention. In other words, these are strategies intended to simplify and strengthen the process of implementing an intervention, serving as the foundation for subsequent integration of a given intervention in a treatment setting. Examples include setting organizational goals for practice change, identifying implementation barriers, and modifying an intervention to improve contextual fit. Within the delivery system, this often takes the form of quality improvement projects, which may utilize implementation process strategies to adapt an intervention to better fit the setting in which it will be delivered.

#### Implementation process strategies in CBT

The Beck Initiative is an example of successful use of implementation process strategies which has produced numerous empirical investigations and reports in addition to increasing access to CBT (29). The Beck Initiative is an academic and public health partnership intended to increase the availability of training and services to the Philadelphia, PA community. The implementation process follows the Assess and Adapt—Convey Basics—Consult—Evaluate—Study Outcomes—Sustain (ACCESS) model of which the first step is *Assess and Adapt* (30). Practically, this step has the training team meet with stakeholders in the organization in which CBT is to be implemented with the goal of increasing engagement. During the early stages of engagement, the trainers work with the providers to determine their organizational goals and conduct a needs assessment to evaluate context-based needs. The training process is then adapted to meet those needs. For example, an outpatient community clinic with limited staffing resources might require a tailored approach that emphasizes a brief course of individual therapy to maximize clinician availability. Alternatively, training clinic providers in group CBT may increase access by increasing the patient-to-provider ratio. This process is closely aligned with the implementation process category delineated by Leeman et al. and although it is specific to CBT in the case of the Beck Initiative, the strategies could be equally applied to any psychosocial intervention.

The most recent reports from the Beck Initiative show that the approach has led to 348 clinicians trained across 42 clinical sites (29). Of those clinicians who initiate the training, nearly 80% completed the training and a comparable proportion reached acceptable fidelity following training. Although data are yet to be published to demonstrate the specific effect on psychiatric symptoms, the weight of evidence indicating the effectiveness of CBT combined with the training outcomes suggests a net positive effect for this approach and highlights the role of implementation process strategies in successful implementation.

#### Critical issues for implementation process strategies in CBT

Attending to the process for implementing an intervention is critical to successful implementation. There are numerous strategies to improve the process of implementation, such as identifying an intervention that is appropriate for the clinical context and adapting or tailoring it to improve fit. For example, a large multi-specialty healthcare facility that would like to increase access to evidence-based mental health interventions for trauma-exposed populations could attempt to incorporate a cognitive-behavioral intervention for PTSD. Prolonged exposure and cognitive processing therapy (CPT) are both candidates for implementation; however, failing to account for a shortage of mental health professionals and poor funding for these positions could lead to inadequate implementation if the organization attempted to deliver one-on-one CPT for the standard 12 sessions. Instead, the organization might consider implementing a group format of CPT to maximize efficiency in a limited resource context. Group format CPT has demonstrated efficacy and thus could overcome clinical capacity barriers (31). Alternatively, delivery of an abbreviated form of prolonged exposure could increase access. Such an abbreviated format has been evaluated though there are no widespread efforts to implement the intervention (32). Additionally, setting goals for the implementation of the intervention will contribute to improved motivation for change and provide a means of evaluating clinical outcomes.

Our attempt to identify examples of implementation process strategies in CBT research and practice yielded few instances where these strategies were employed and documented. We suspect that a primary reason that implementation process strategies were used less frequently than expected is that attending to readiness for implementation may not be a typical practice in these trials. Accordingly, employing strategies to improve the readiness and process of implementing interventions is not as common. Leeman et al. suggest establishing goals and objectives as one of several implementation process strategies, which parallels the work of CBT providers and other mental health professionals who are trained to obtain patient goals and objectives for treatment. As such, the parallel is apparent for those attempting to implement CBT wherein identification of goals and objectives coupled with targeted interventions (i.e., implementation process strategies) could improve readiness for implementation.

Many clinical trials of CBT interventions likely utilize implementation process strategies in the design or execution of the study procedures, but do not specify these in research reports. Indeed, our attempt to identify exemplars to illustrate this category was challenged by the presence of relatively few studies. This may have been a consequence of authors not specifying implementation process strategies in their reports more so that not actually using them. If so, this is a limitation to the CBT literature because it could imply limited efforts to lay the foundation for implementation instead of simply not identifying these in the resulting manuscript. Indeed, we identified several trials that would have made excellent examples, but did not specify a given implementation strategy even if it seemed one was used. Including details about the implementation process in CBT studies could have the positive effect of assisting subsequent researchers to expedite the translation of research to practice. CBT researchers and implementation teams could follow the guidelines for documenting implementation strategies to improve reporting of what strategies they use to improve the implementation of their interventions (12).

### Integration strategies

Integration strategies focus on changes at the level of the clinics, treatment teams, or individual actors within the inner setting of a health delivery system. They are tools that facilitate use of what works by the unit providing a service to move an innovation into the healthcare setting. This is best achieved through identification of barriers and strategies to overcome these barriers in a specific clinical setting.

Integration strategies can be as simple as setting reminders in a clinical documentation program or electronic health record to prompt clinicians to employ a specific clinical technique (e.g., exposure) or the timing of an intervention within a larger EBP package (e.g., completion of an impact statement in CPT). They can be more complex as well, such as using an intensive audit and feedback system to monitor and review the consistency and fidelity with which an intervention is employed. In sum, these are practical strategies that are used to increase implementation of a specific intervention in a specific setting. A limitation of these integration strategies is that they may require technological or other resources that some settings lack. Thus, integration strategies cannot be considered one-size-fits-all, and instead likely requires attention to current system resources and capacity.

#### Integration strategies in CBT

We identified a process developed by Sawchuk and colleagues aimed at improving utilization of empirically supported principles of change in treatment of mood and anxiety disorders in primary care as a prime example of integration strategy use in practice (33). In this example, the authors modified their medical record system to facilitate simple identification of a therapeutic principles used in a psychotherapy encounter. Clinicians were trained to document the principles used in the encounter while completing clinical documentation following service delivery. They were presented with a list of options that providers could check off in the platform. These responses could then be extracted to evaluate the degree to which providers are engaged in EBP and provide feedback to providers about intervention use. This provides a clear example of the potential for integration using technological advances to facilitate integration of CBT interventions.

#### Critical issues for integration strategies in CBT

Integration strategies are a domain of implementation strategies well aligned with the strengths of CBT researchers and intervention developers. Efforts to wed technology and CBT interventions, such as through the use of apps and other mobile technologies, is a broader example of the use of integration strategies. The net effect of this is improved integration and increased availability of effective interventions for patients. It seems apparent that there is capability on the part of intervention developers to use technology to improve access to care, though it is less obvious that it is routinely used to truly integrate an intervention into a specific clinical setting. Indeed, for all the efforts to develop, validate, and disseminate technological interventions, the degree to which these become part of the routine clinical operations of a clinical setting is unclear. In addition to developing technology-assisted interventions, the field could place great emphasis on optimizing integration through technology (e.g., improvements in medical records systems).

An increasingly used integration strategy is task-shifting in which new care teams are developed by way of expanding the types of providers and care teams beyond the traditional world of psychologists and psychiatrists (34). An advantage of CBT is that it is highly scalable in terms of being more easily taught to trainees without requiring advanced training in a mental health profession. Several research teams have undertaken the task of demonstrating the capability of entry-level and lay providers to deliver CBT, sometimes with effects comparable to those of experts (35–38). This further highlights the fit between integration strategies and CBT. A limitation of this work is that authors frequently do not note the aim of these efforts, which is in our view to increase the *integration* of CBT into the intended clinical setting, rather than to simply increase the number of providers or access to care.

### Capacity-building strategies

Leeman et al. define capacity-building strategies as those “delivered by support systems and target individuals’ general capacity to execute implementation process strategies.” The aim of a capacity-building strategy is to provide people with the training and tools they need to be able to complete best practices for implementation. It is separate from the training in the intervention itself and focuses squarely on the implementation skills needed to move the intervention into practice within the chosen setting.

A simple example of a capacity-building strategy is deploying a team to train clinic staff in the Plan-Do-Study-Act cycle of quality improvement with the goal of developing the clinic’s *capacity* to solve problems that might later occur during intervention implementation (39). Ongoing technical assistance to support the continuation of quality improvement initiatives would also be consistent with capacity-building strategies. In sum, a strategy intended to increase the capacity of an individual or organization to implement CBT (or any other EBP) would be considered a capacity-building strategy and is analogous to an intervention intended to improve an individual’s ability to put into practice behavior change.

#### Capacity-building strategies in CBT

Capacity-building strategies applied to CBT would include efforts aimed at improving an organization’s readiness to engage in implementation of CBT, rather than training in capacity to conduct CBT specifically. The Evidence-Based Practice and Innovation Center (EPIC)^1^ currently in place within the Philadelphia behavioral health system provides an example of this process (40, 41).

An organization intending to implement an EBP with guidance from EPIC begins with an application process in which the organization applies to be an EBP Initiative within the larger system. The application process encourages the organization and its leadership to learn and plan for implementation before the actual implementation work begins. Upon successful application, the organization works with an implementation consultant as part of a group of providers and organizations with the responsibility of helping the organizations improve their readiness to implement the interventions using the application materials as a foundation. The consultant engages in a number of activities to build a general capacity to implement an EBP, incentivize sustainable financial structures, planning for viability and sustainability post-implementation, and generally improving the implementation plan. These strategies continue in the form of quarterly meetings to support application of implementation processes (e.g., facilitating development of community relationships) as the organization works to implement the intervention.

Importantly, technical assistance in implementation processes occur across the implementation timeline through the use of a web-based tool and ongoing webinars hosted through EPIC, with more frequent use prior to implementation to prepare the general capacity for implementation. The web-based tool and webinars build motivation for implementation, aid in understanding critical issues in implementation (e.g., determining organizational “fit” with available interventions), and deliver updated guidelines on implementation of selected interventions. This form of technical assistance is similar to what might be expected in the scale-up of a specific EBP within a given clinical setting; however, the technical assistance employed by EPIC aims to improve the general capacity to engage in implementation processes rather than the EBP per se.

The EPIC approach resulted in increased availability of several EBPs within the larger CBT framework, including cognitive therapy, prolonged exposure, and dialectical behavior therapy, among others (42). Although the authors reported only “modest” increases in utilization of EBPs supported through EPIC (e.g., 6% increase in self-reported EBP use), such a change has enduring effects within a sizable provider base delivering care to a large population and has the potential for downstream influence on other providers within the system. That is, even a small self-reported increase in the frequency of CBT technique usage increases EBP exposure, and potentially decreases use of practices that lack a strong evidence base. An expansion of the EPIC approach to additional mental health systems would further expand the reach of these interventions and may identify additional moderators of effect of capacity building.

#### Critical issues for capacity-building strategies in CBT

Ultimately, the outcome of capacity-building strategies is improving individuals’ ability to implement an EBP using implementation process strategies with demonstrated effectiveness, such as training, technical assistance, and networking/coaching. It is clear that providing a single training in a specific EBP within the complex and dynamic context of mental health is insufficient. Instead, working from the ground up by training a clinic or larger system in implementation processes is necessary to achieve widespread, sustainable implementation of a given EBP.

A consistent barrier to capacity-building strategies within mental health is a lack of time and resources. As a provider, supervisor, or administrator, engaging in capacity building takes time, and the immediate benefit of capacity building may not be evident. These stakeholders are more likely to view the training in the EBP as the critical step, ignoring the need to “set the stage” for implementation. The result is often training in an EBP with potential but without the supports for implementation and ability to use these supports, followed quickly by decreased utilization of the EBP and a return to the status quo. We suspect that observations of EBPs ineffectively implemented contributes to the belief among some providers that EBPs (i.e., research-based interventions) do not work because they failed to deliver the expected effects. Indeed, the belief that EBPs do not work is one of the recognized barriers to willingness to engage in EBP more broadly (43). Alternatively, the absence of institutional support for EBP implementation may produce resistance or, at minimum, lack of motivation to EBP use, given evidence that such support is imperative for effective implementation (44).

### Scale-up strategies

According to Leeman et al., scale-up strategies employ support systems to implement an EBP across multiple settings. Scale-up strategies target multiple levels: individuals, inner setting, and outer setting. Scale-up strategies focus across action levels and may employ a variety of approaches to improve the capacity to integrate an EBP across multiple settings. Approaches may include training, technical assistance, implementation toolkits, benchmarking, infrastructure development, and service payment.

#### Scale-up strategies in CBT

The large-scale implementation study conducted by Karlin and colleagues provides an ideal example of how to employ scale-up strategies and the scale-up process more generally (9). In this project, the study team trained 15 mental health professionals to deliver a CBT for depression protocol to primary depression patients. The intervention consists of largely standard cognitive-behavioral interventions; however, the study team constructed the training process with the goal of increasing engagement from clinicians by making the learning process more individualized. The individualization process included personalized training goals developed by the clinician in consultation with the trainers. The trainers provided feedback throughout the training and collaboratively revised the goals as needed. Additionally, the training program was experiential to increase active engagement with the material and promote acceptance of CBT on the part of the trainee clinician. These modifications to training are predicated on the hypothesis that increasing “active learning” will increase willingness to provide CBT for depression.

Importantly, the intervention also emphasized feedback provided by the clinician in the course of intervention. As the authors state, “The significant focus on feedback incorporated into the Kaiser-Permanente (KP) CBT-D protocol aligns with and is an important part of a larger initiative to advance feedback-informed care as a key priority within the KP behavioral health care system” (p. 448). Thus, the decision to include an emphasis on feedback-based care is consistent with an existing institutional standard. This is an example of tailoring materials to more effectively match the culture of the organization and thus increasing the likelihood of successful implementation. This approach contrasts with implementation efforts that disseminate information without considering organizational culture. With 100% of trainee clinicians reaching a point of CBT competence following the training, the resulting data suggest that this method of disseminating information to clinicians outperforms similarly intense training programs. The effects were not limited to CBT skills, as the effect of training was also observed on other common factors of therapeutic effect. Notably, the structure and function of training closely mirrored that of the intervention itself, which emphasized individualization and feedback during delivery of the intervention.

#### Critical issues for scale-up strategies in CBT

Engaging a system to scale up an intervention is challenging and requires buy-in from multiple stakeholders and across organizational levels (front line, leadership, administration), and is strengthened by including those who are from the outer setting and may not be closely identified with the work. It may also require engagement across organizations with different disciplinary styles, missions, funding streams, and deliverables. Of course, it may not be feasible for all stakeholders at all levels to be engaged in all aspects of implementation. In these instances, providing focused implementation support to those aspects that are most relevant to each group is an effective approach.

Alignment across implementation activities, policies/regulations, and payment facilitates sustainable solutions. Individuals and organizations must be allowed to conduct the EBP and implementation process strategies to support it – that is, the activity needs to be accepted within policies and regulations. Further, individuals and organizations must be paid for these activities, otherwise it is not feasible to deliver them. For example, one approach to address mental health workforce shortages is peer delivered evidence-based interventions. For this to be a sustainable solution, policies and regulations must allow peers to conduct this work and provide a framework for scope of service and oversight. Financing would need to address payment of peers and those who supervise them as well as payment for documentation, training, and implementation support.

## Hypothetical implementation design using Leeman categories

We have provided examples of how existing cognitive-behavioral research has utilized the broad implementation strategy categories developed by Leeman and colleagues. Importantly, and as is evident in several of the examples described above, these strategies are not mutually exclusive. Indeed, the most successful implementation efforts will likely utilize several or even all of the strategy categories to achieve the implementation aims. As such, we offer the following *hypothetical* implementation study design to evaluate the implementation of a brief form of exposure and response prevention (ERP) for OCD. We selected ERP because it has remarkable efficacy, but also relatively low penetration in community-based treatment settings (45).

### Dissemination strategy

The implementation team developed a 4-session abbreviated version of ERP intended for delivery in low-resource intensive settings and piloted the intervention in several small studies to refine the content and test its efficacy. After demonstrating a moderate effect in a sufficiently powered effectiveness trial, the team proposed to test its implementation in a cluster-randomized trial of community-based outpatient clinics. Prior to soliciting involvement from the clinics, the team conducted an evaluation of the marketing content of the intervention to determine what descriptors of the intervention were most appealing to the target audience of social workers in the relevant clinics. They then used these descriptors to modify the messaging about the intervention. For example, they noted that rather than the term “psychoeducation” in the intervention materials, social workers preferred to call this process “sharing information.” Similarly, they suggested abandoning the term “brief exposure and response prevention” and replacing this with “OCD Coaching.” Accordingly, the implementation team modified their materials to reflect these suggestions. Finally, the surveyed clinicians advised the team to use local, community-based therapist listservs to disseminate information about the intervention in an effort to more effectively engage clinicians in the project. They also identified several social media outlets with large representation of the intended clinical workforce and targeted dissemination through these channels.

### Implementation process strategy

Following development of materials to disseminate information to study sites and their clinicians, the implementation team met with administrators and clinicians at six clinics to increase engagement, determine potential barriers to implementing the abbreviated ERP intervention, and identify strategies that they believed would increase the likelihood of successful initiation and sustainment of the intervention. The respondents cited a need for flexible training schedules given the clinical workloads along with a general need to better understand the goals of implementing the intervention. They also stated that revising documentation templates would likely improve their use of the intervention and that ongoing consultation with a content expert would increase confidence in their clinical skills. The team modified the implementation approach to reflect the recommended changes shared by these sites.

### Integration strategy

The implementation team developed a series of documentation templates that were specific to the electronic medical record systems of each clinic, but included the essential elements of brief ERP. They also dedicated one consultant for each of two clinics to serve as a content expert who would review 10% of all clinical documentation to evaluate fidelity to the intervention. The consultants led eight weekly, hour-long group supervision sessions to provide clinical guidance for implementing the intervention across the site pairs to facilitate cross-clinic learning. This process was intended to allow for 1–2 full courses of the intervention over the 8-week interval. They were also available for brief phone consultation as needed.

### Capacity-building strategy

Efforts to increase the capacity of the sites to implement the intervention initially occurred prior to integration of the intervention, but continued throughout the trial. The goal of capacity building efforts in this context were to support clinical leadership and clinicians to engage in quality improvement processes, including monthly coaching sessions focused on relevant topics such as clinical outcomes tracking, sustainable reimbursement models, and site-specific adaptation procedures. The team trained these sites in the Plan-Do-Study-Act quality improvement process as an initial step to preparing them for implementation and clinical outcomes monitoring (39).

The implementation team assigned a co-investigator to act as an external facilitator who monitored the clinical personnel’s use of the intervention and skills learned from the preparatory implementation training. They also reviewed the clinics’ patient enrollment logs to identify any appropriate patients that did not receive the intervention during the study period and conducted an analysis of missed patients to determine what process gaps led to the patient not receiving the intervention. Importantly, they met monthly with the clinic administrators to review consistency in implementation and trained them in the auditing procedures to support eventual independence of implementation.

### Scale-up strategy

In response to the initial survey recommendations and meetings with clinic staff, the intervention training was conducted online using a flexible schedule, followed by computerized evaluations of knowledge of the intervention. The program could be completed with variable timing so long as all training sessions were completed within a two-week interval. This was included as one component of a larger implementation toolkit that was agnostic to the specific clinic and adaptable to clinics of varying size and clinician compositions (e.g., psychologists, social workers, etc.). Additionally, the implementation team selected one clinician from each study site to comprise an implementation workgroup that met quarterly to review implementation from the clinicians’ perspective and identify areas of need for improvement across the network of clinics.

## Conclusion

CBT has partially benefitted from dissemination and implementation efforts; however, the focus of these efforts has left gaps that may limit access to services for patients and contribute to inconsistent uptake among frontline providers. We suggest that increasing knowledge that CBT is effective through dissemination efforts is a necessary step in improving implementation of the intervention, but it is insufficient for implementation. An equally important component alongside dissemination strategies is the inclusion of implementation strategies in the research demonstrating effectiveness and, of course, actual implementation trials. Lines of clinical research that do not extend beyond efficacy or examine implementation strategies within effectiveness research represent missed opportunities to translate innovation into action.

The studies described in this narrative review all utilize one or more implementation strategies align with the Leeman categories. These are largely effectiveness, implementation, or quality improvement projects in practical clinical settings led by researchers trained in implementation science methods. It is not surprising that researchers trained in the science of dissemination and implementation would not only utilize these strategies, but would also specify them in some way. Importantly, however, being an effective CBT researcher and intervention developer does not require expertise in implementation science methods any more than being an implementation scientist studying CBT requires expertise in delivering an intervention. A method to increase the implementation of CBT practices may include increasing the use of methods borrowed from implementation science and documenting the strategies employed by the research teams. The examples highlighted above used many of these strategies, though variable documentation of the strategies may decrease communication of what works in dissemination and implementation. Clearer identification and description of the implementation strategies would likely improve the communication and consistency across studies and clinical targets of those studies.

### Barriers to applying implementation categories

We initially sought to conduct a scoping review of CBT studies that utilized strategies corresponding to the five categories identified by Leeman et al. We quickly realized that many studies did not clearly identify strategies used or mislabeled strategies (most commonly referring to implementation as dissemination or vice versa). We identified relatively few clear exemplars implementation process or capacity-building strategies. Thus, we revised the goal of this effort to serve as a narrative review to describe the application of implementation strategies to CBT research and practice using the Leeman et al. categorization system as a guide rather than an exhaustive review of the literature on implementation science applied to CBT. As such, this narrative review has several important limitations to specify, including the non-exhaustive nature of purposive literature sampling, interpretive nature of exemplar selection and categorization, and potential for studies to fit as exemplars in multiple categories.

Regarding the use of purposive literature sampling, we employed a simplified search process using selected terms that could reasonably approximate the descriptions of the categories and their intended functions (e.g., using “integration” alongside “setting” or “support” to identify exemplar studies of Integration strategies). Thus, there may be other exemplars of the strategy categories that were not identified using this approach. This is a limitation that would need to be addressed in a true scoping review, but it also highlights the value of using a common language to categorize strategies.

The selection of exemplars for a specific categorization that is not commonly used also has obvious limitations. Because the Leeman categories are only one system for organizing strategies, the process used to identify exemplar studies hinged on recognizing the strategies included in each report as belonging to the categories based on those category criteria, specifically the ISF actor type and CFIR target domain. As a result, many published studies may have used strategies that could be grouped within those categories, but were not selected as exemplars because the ISF or CFIR domains were unclear. This may also provide some encouragement for utilizing the Leeman categories to efficiently summarize and conceptualize the strategies employed to aid in dissemination and implementation of CBT in routine practice based on actor and target specification. Furthermore, including a description of the strategies, consistent with the recommendations of Proctor et al., would be as beneficial a practice as the specification of any other aspect of study methods reported in manuscripts.

Finally, several of the studies described here utilized strategies from multiple categories, consistent with the understanding of implementation as a multifaceted effort that spans the types of strategies depicted in this manuscript. Thus, the process of consensus for which studies served as exemplars was subjective, aided by the Leeman category criteria. Certainly, inclusion as an exemplar for one category does not negate its relevance for other categories. Indeed, implementation mirrors the therapeutic process in many ways wherein an effective intervention is comprised of several active “ingredients” rather than one strategy that carries the full effect of the intervention.

Importantly, we acknowledge that many studies evaluating EBP implementation efforts, including those reviewed here, focus on delivery of the EBP in large systems and often those with more resources than what might be found in smaller organizations or independent practice settings. Greater emphasis on evaluating implementation efforts in small, independent clinical settings where a substantial proportion of mental health providers deliver care is an important effort that has not yet been attempted at scale. It is also possible that efforts to improve dissemination, implementation processes, integration, capacity building, and scale-up of intervention may still fail to meaningfully or consistently improve clinical outcomes. There are other determinants of clinical outcome that are not addressed by all of the categories of strategies including in this model, but these categories are nonetheless an important framework for understanding the armamentarium of strategies aiming to effect health outcomes.

## Footnotes

1
https://dbhids.org/epic/
